# Autistic adults exhibit holistic face processing: evidence from inversion and composite face effects

**DOI:** 10.3389/fnins.2024.1393987

**Published:** 2024-11-12

**Authors:** En-Yun Hsiung, Sarina Hui-Lin Chien

**Affiliations:** ^1^Graduate Institute of Biomedical Sciences, China Medical University, Taichung, Taiwan; ^2^Center for Neuroscience and Brain Diseases, China Medical University, Taichung, Taiwan

**Keywords:** face perception, composite face effect, face inversion effect, holistic processing, autism spectrum disorders

## Abstract

Holistic processing is commonly measured by the face inversion effect (FIE) and the composite face effect (CFE). Previous studies examining whether individuals with autism spectrum disorder (ASD) employ holistic processing using either FIE or CFE have reported inconclusive results. By adopting a customized composite face paradigm, the present study aims to simultaneously assess both the inversion and the composite effects of holistic processing in autistic and neurotypical adults. We tested 24 adults with ASD and 24 neurotypical (NT) adults matched in age, gender, and years of education. Participants viewed sequentially presented composite faces in three Presentation Modes (*aligned*, *inverted*, and *misaligned*) with three Stimuli Conditions (same, composite, and different) and judged whether the top half was the same. For the dependent variables, we calculated a “*performance index*” in the form of the accuracy/response time of each stimuli condition in each presentation mode. The FIE and CFE were computed to index the magnitude of holistic processing. Our results showed that the NT group responded more accurately in less time than the ASD group across task conditions. Notably, both the NT and the ASD groups exhibited a significant FIE with similar magnitude. Likewise, both the NT and the ASD groups showed a greater-than-zero CFE. Moreover, individuals’ CFE positively correlated with FIE and negatively correlated with the AQ scores for all participants. In summary, individuals with ASD exhibit holistic processing when viewing faces, evidenced by the presence of both FIE and CFE and the positive correlations between the two effects.

## 1 Introduction

Autism spectrum disorder (ASD) is a neurodevelopmental condition characterized by persistent deficits in the ability to initiate and sustain reciprocal social interaction and communication, and by a range of restricted, repetitive, and inflexible patterns of behavior, interests, or activities that are atypical or excessive for the individual’s age and sociocultural context (World Health Organization, 2018). Face recognition is essential for social interaction and communication. Although aberrant face processing is not a core clinical feature for diagnosing ASD, many studies reported behavioral abnormalities with face processing in ASD (for a review, see Behrmann et al., 2006; Weigelt et al., 2012; Tang et al., 2015; Tanaka and Sung, 2016). For example, autistic individuals have been reported to have difficulties in tasks involving discrimination of facial identities (Tantam et al., 1989; Behrmann et al., 2006; Wallace et al., 2008; Hadad et al., 2019; Hsiung et al., 2022), recognition of familiar faces (Boucher and Lewis, 1992), immediate recognition of novel faces (Blair et al., 2002; Boucher and Lewis, 1992; Gepner et al., 1996; Klin et al., 2002; Faja et al., 2008; Joseph et al., 2008; Wilson et al., 2010; Chien et al., 2014) or within-person face recognition (Neil et al., 2016).

Parallel with the behavioral findings, fMRI studies reported aberrant neural activities during face processing in individuals with ASD (Pierce et al., 2001; Schultz et al., 2000; Hubl et al., 2003; McCleery et al., 2009; Okamoto et al., 2017). Schultz et al. (2000) revealed that during face (but not object) discrimination, adults with ASD engaged the inferior temporal gyri (i.e., an area known for object perception in neurotypical adults) significantly more than the healthy controls, demonstrating a pattern of brain activity consistent with feature-based strategies that are more typical of non-face object perception. Pierce et al. (2001) reported that participants with ASD showed a pattern of individual-specific, scattered activation when viewing faces, whereas neurotypical controls exhibited a highly consistent fusiform gyrus activation. A recent fMRI study further endorsed these findings by showing that children with ASD exhibited atypical activation in the fusiform face area (Okamoto et al., 2017), the core brain loci involving with the structural encoding of faces (Haxby and Gobbini, 2011).

Holistic face processing, a cornerstone of the configural process, is the tendency to glue facial features together as a gestalt (Maurer et al., 2002). As opposed to object processing, which is more feature-based and part-based (Tanaka and Farah, 1993), holistic face processing involves a mandatory perceptual integration across the whole, including precise spatial-relational information among facial features (McKone et al., 2007). Evidence for holistic processing comes from two kinds of convincing demonstrations: the *face inversion effect* (FIE) and the *composite face effect* (CFE). The face inversion effect (Yin, 1969; Hole, 1994) refers to performance decrement for upside-down faces compared with upright faces (McKone et al., 2007), typically computed as FIE = (performance of upright) – (performance of inverted). Although part-based processing is still preserved for inverted faces, holistic processing is substantially impaired by inversion (Rossion, 2013). The composite face effect, first demonstrated by Young et al. (1987), refers to a compelling phenomenon that “when the top half of one face was aligned with the bottom half of another, the resulting composite induced the perception of a novel facial configuration. Therefore, participants are often slower and less accurate in recognizing the top half of one face presented in a composite with the bottom half of another face when the composite is upright and aligned, as opposed to when the two halves are misaligned (Young et al., 1987; Murphy et al., 2017). The misalignment of the face allows a person to ignore the bottom half, which is difficult to do when the face is aligned. Therefore, when a person process faces holistically (considering both the top and bottom of the face), they perform better when they can ignore the bottom (i.e., perform better when the face is misaligned rather than aligned). On the other hand, if a person uses a feature-based approach, they may be better at ignoring the bottom half of the face even when it is aligned (Maurer et al., 2002; Cheung et al., 2008; Richler et al., 2009; Rossion, 2013; Murphy et al., 2017).

Although the reasons for face-processing difficulties in ASD remain unclear, it is suggested that the atypical development of face-processing may result from reduced social interest or pervasive perceptual atypicalities (e.g., Behrmann et al., 2006). One such perceptual atypicality is the difficulty of using holistic processing; they tend to encode and represent visual information on a local, part-by-part basis rather than holistically (Behrmann et al., 2006; Pallett et al., 2014; Hsiung et al., 2022; cf. Tanaka and Sung, 2016). Pallett et al. (2014) used a morphing paradigm to investigate the discrimination sensitivity of faces and objects in adolescents with ASD and their TD peers. The ASD group showed slight impairments in discrimination sensitivity for faces yet significantly enhanced sensitivity for objects, supporting the feature-based processing bias in ASD.

This difficulty with holistic processing is further supported by studies reporting an absence or a reduced FIE in ASD. It is widely accepted that the FIE arises from a shift in encoding style, with upright faces being encoded more holistically and inverted faces encoded feature-based (Farah et al., 1998; McKone and Yovel, 2009; Young et al., 1987). Several studies claimed that individuals with ASD still engage in holistic processing that their FIE was not qualitatively different from that of neurotypical controls (Bar-Haim et al., 2006; Lahaie et al., 2006; Rutherford et al., 2007; Teunisse and de Gelder, 2003; Joseph and Tanaka, 2003). However, other studies reported a significantly smaller or atypical FIE in autistic individuals (Rose et al., 2007; Nakahachi et al., 2008; Hadad et al., 2019; Hartston et al., 2023). Nakahachi et al. (2008) presented pictures of a normal or a Thatcherized face side-by-side upright or inverted and asked participants to make the same/different judgment. The ASD group exhibited significantly longer response times in the upright condition but not in the inverted condition, indicating a deficit in holistic processing. Using a delayed estimation task in which a single target face was shown either upright or inverted, Hartston et al. (2023) reported that the ASD group made more errors than the TD group in both the simultaneous (i.e., encoding process) and delayed (i.e., mnemonic processing) intervals. The ASD group exhibited weaker FIE than the TD group on all retention intervals, suggesting that weaker face recognition deficits in ASD arise from perceptual-based alterations.

The composite face effect is another direct way to measure holistic processing (Young et al., 1987; Maurer et al., 2002; Cheung et al., 2008; Rossion, 2013). A handful of studies investigated composite face effects in ASD and reported discrepant results. Using both a face inversion task (Exp.1) and a composite face task (Exp.2), Teunisse and de Gelder (2003) compared the performance of three groups: high-ability adolescents with ASD, TD children, and adults. They found that the size of FIE in the adolescents with ASD was not significantly different from that of TD children and adults, indicating that the ASD group still forms a configuration-based face representation. However, the ASD group recognized face halves equally well in the upright-aligned and the misaligned conditions; the absence of the typical composite effect suggests deviant holistic processing in the ASD group. It shall be noted that the interpretation of Teunisse and de Gelder (2003) may be dependent on age as well, as different age groups were compared. On the other hand, Nishimura et al. (2008) found that adults with ASD, like age- and IQ-matched controls, demonstrated a typical composite face effect that the holistic interference was present in the aligned condition (and not the misaligned condition).

To overcome the limitations of Teunisse and de Gelder (2003) and Gauthier et al. (2009) employed the “complete composite design” and tested adolescents with ASD and healthy controls well-matched on sex, age, and IQ. Like the neurotypical controls, adolescents with ASD also experienced interference from facial features that they were told to ignore. However, when parts of face composites were misaligned, the adolescents with ASD showed comparable interference from irrelevant parts regardless of alignment, suggesting a qualitative different holistic processing and a role for attentional abnormalities. Two recent studies adopting the “Complete Composite Face Task” to test autistic adults have revealed a “normal” or a “qualitatively similar” holistic processing. Dunsworth (2016) found that holistic processing is evident in TD children and autistic adults but not in autistic children, suggesting a developmental delay in holistic processing in children with ASD. Ventura et al. (2017) found that autistic adults process faces holistically and their abilities are qualitatively similar to those of neurotypical adults. Although it remains inconclusive whether autistic individuals exhibit a “qualitatively normal” or an “atypical” holistic processing, these collective findings seem to suggest an important role for age. However, both Teunisse and de Gelder (2003) and Gauthier et al. (2009) did not include a group of adults with ASD in their studies, which might have revealed whether holistic processing becomes more typical (i.e., only quantitative difference) by adulthood or whether holistic processing remains qualitatively different in ASD (Ventura et al., 2017).

Holistic processing is the hallmark of human face perception; our ability to recognize and differentiate between faces relies heavily on perceiving/processing them holistically, as all faces have an inherently similar structure (e.g., Richler and Gauthier, 2014). As illustrated above, the *face inversion effect* (FIE) and the *composite face effect* (CFE) are the most convincing demonstration of holistic processing. However, up to date, only Teunisse and de Gelder (2003) tested individuals with and without ASD using both a face inversion task and a composite task simultaneously, and their results were puzzlingly intermixed; the ASD group showed evidence for holistic processing on the face inversion task, but not on the composite face task. Moreover, it was difficult to rule out the confounding factor of age because the ASD group (adolescents) and the TD groups (children and adults) did not match in biological age. Hence, the present study aims to investigate holistic face processing in Taiwanese adults with ASD and age-, gender-matched neurotypical controls (NT). Using a customized partial composite face paradigm to simultaneously assess both the inversion effect (FIE) and the composite face effect (CFE), we intend to validate whether autistic adults exhibit qualitatively similar holistic processing as their non-autistic peers from convergent evidence. To achieve this goal, we adopted three Presentation Modes (*aligned*, *inverted*, *and misaligned*) on three Stimuli Conditions (same, composite, and different) for only the top face (see Gauthier et al., 2009). For the dependent variables, we took both accuracy (ACC) and response time (RT) into account by calculating a “*performance index*” in the form of ACC/RT (in seconds) of each stimuli condition in each presentation mode (see Rossion, 2013). This performance index will subsequently be used to analyze the magnitude of holistic face processing.

## 2 Methods

### 2.1 Participants

The participants comprised 24 autistic adults and 24 neurotypical adults matched in gender (ASD group: 13 men, 11 females; NT group: 13 men, 11 females) and age (ASD group: 28.93 ± 5.40 years; NT group: 28.19 ± 5.20 years). This sample size was calculated according to Rosner et al. (2005) and was determined based on power consideration (i.e., we set the type 1 error (*α*) to 0.05, the power (1-*β*) at approximately 0.84, leading to an expected sample size of 24). The autistic adults were recruited via the Taiwanese Asperger’s Club from a private Facebook group, primarily from the Taipei and Taichung metropolitan areas. Neurotypical adults were recruited via the CMU Facebook student club; they were primarily undergraduate or graduate students of China Medical University, Taichung, Taiwan. The education level of both groups was well-matched (ASD: *M* = 16.13 years, SD = 1.42, NT: *M* = 15.96 years, SD = 1.76), which was calculated by the years of education (i.e., with high-school diploma = 12 years; with bachelor’s degree = 16 years; with postgraduate educations >16 years). Written informed consent was obtained before the experiment. The protocol of the present study was approved by the Human Research Ethics Committee, China Medical University Hospital, Taichung, Taiwan (The IRB certificate: CMUH103-REC3-055). All participants had normal or corrected-to-normal vision (20/20) and self-reported with no history of visually related problems. Table 1 summarizes the characteristics of the groups. Participants in the ASD group were clinically diagnosed with DSM-IV-TR (American Psychiatric Association, 2000) or ICD-10 (World Health Organization) in their childhood or adolescence as having Asperger’s syndrome or high-functioning autism by physicians in government-appointed hospitals. Their diagnosis was based on standard instruments such as ADOS-2 and ADI-R. Notably, most of the participants in the ASD group held an official “Type 1 Disability Card” (with nervous system dysfunctions and mental and psychiatric diseases) issued by the Social Welfare Department of the city government. All participants in the NT group were retained in the final sample, and each had an AQ score below the cutoff criteria. One additional participant in the ASD group was tested but excluded from the final sample because of a low AQ score of 23. Each participant received a cash payment and traveling expense compensation if needed.

**Table 1:** Summary of the group characteristics.

	ASD group (*N* = 24)	NT group (*N* = 24)	*p* value
Gender (M:F)	13:11	13:11	
Age (yrs)	28.93 ± 5.40	28.19 ± 5.20	0.631
Education (yrs)	16.13 ± 1.42	15.96 ± 1.76	0.720
AQ score	38.17 ± 5.72	18.83 ± 5.57	<0.001***

Error bars represent the standard errors of the means (***p < 0.001). AQ, Autism Quotient.

### 2.2 Stimuli, apparatus, and procedure

The participants performed the tasks individually in a quiet Laboratory room. After the experimenter introduced the tasks, the participants filled out the Chinese version of the AQ questionnaires first and then performed the computerized Composite Face Task.

#### 2.2.1 The Chinese AQ questionnaire

Both groups of participants underwent the assessment of the Autism Spectrum Quotient (AQ). We adopted the Chinese pencil-and-paper AQ questionnaire (Liu, 2008), which included 50 questions assessing five dimensions: *social skill*, *attention switching*, *attention to detail*, *communication*, and *imagination*. The total score ranges from 0 to 50, with higher scores indicating higher autistic traits. The mean AQ score of the ASD group (*M* = 37.56, SD = 6.37) was significantly higher than that of the NT group (*M* = 18.85, SD = 5.41), *t*(49) = 11.287, *p* < 0.001. The ranges of AQ scores for the NT and the ASD groups were 11 to 28 and 27 to 48, respectively.

#### 2.2.2 The composite face task

A laptop computer (Acer Aspire Model N16Q2) with a 15.6″ monitor and E-Prime Professional 2.0 (Psychological Software Tools, Sharpsburg, PA) were used to run the *Composite Face Task*. Each participant sat on an adjustable chair allowing his/her eyes to fixate on the center of the monitor with a viewing distance of about 30 cm.

##### 2.2.2.1 Face stimuli

**Figure 1:**
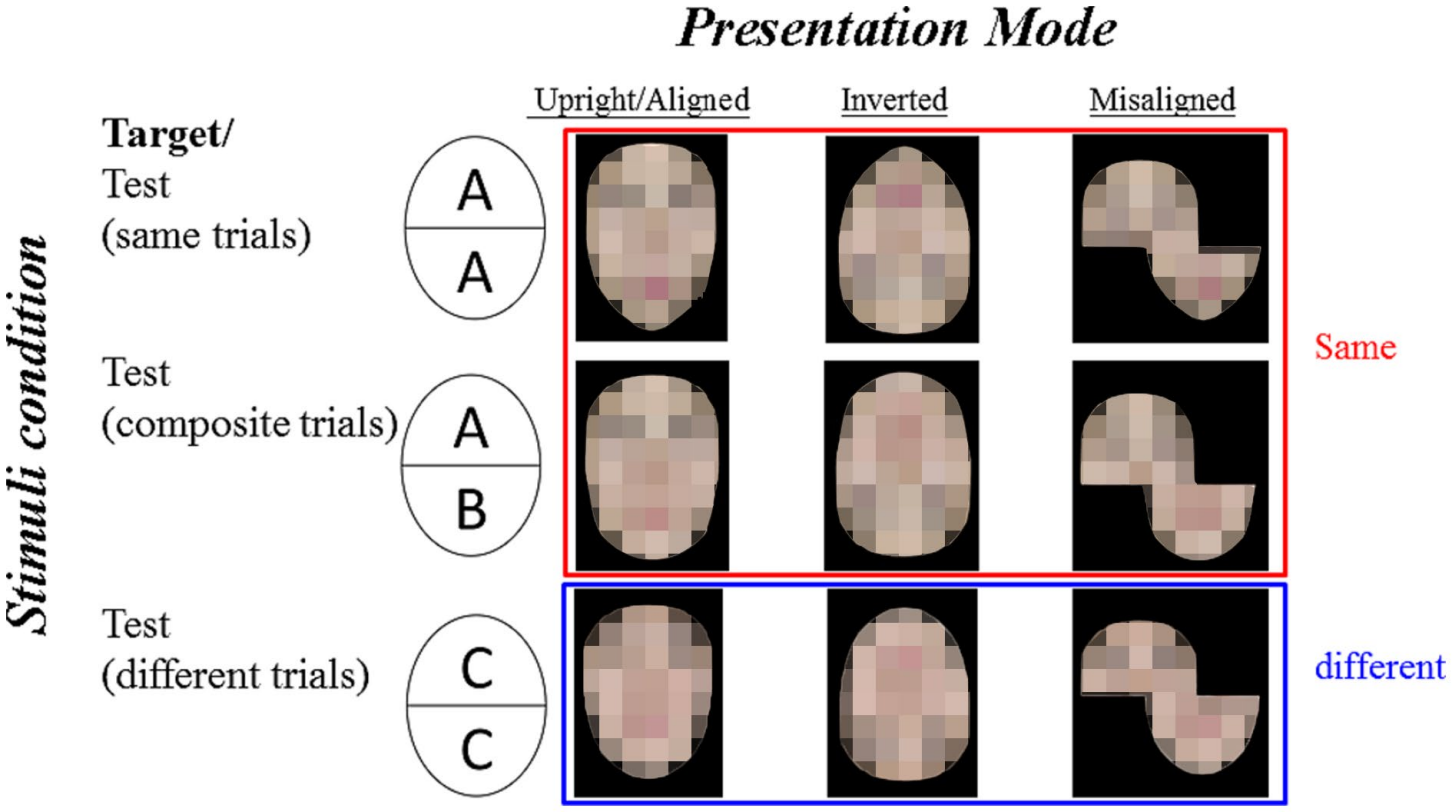
Illustration of sample stimulus examples for the composite face task. For the same trials (identical top and bottom), the target face is AA, and the test face is AA. For the composite trials (identical top with different bottom), the target face is AA and the test face is AB, which is the most interesting type of trials. For the different trials (different top and bottom), the target face is AA and the test face is CC. The three presentation modes are aligned, inverted, and misaligned (from left to right). The correct answers for the same and the composite trials shall be “same” (shown with a red frame) while the correct answers for the different trials shall be different (shown with a blue frame). The identifiable images were blurred to protect the portrait copyright.

The stimuli consisted of eight sets of faces with neutral expressions selected from the Taiwanese face stimuli set (Cheng et al., 2016). Each set included three presentation modes (i.e., *aligned*, *inverted*, and *misaligned*) and each with three stimuli conditions (i.e., same, composite, different). Figure 1 illustrates a sample set of the composite face stimuli. We adopted the method suggested by Rossion (2013), in which a small gap was introduced between the two halves. The size of the *aligned* and the *inverted* faces were about 6 cm (width) x 8 cm (height). The bottom faces in the *misaligned* condition were shifted 50% to the right, and thus the overall size of the *misaligne*d faces was 9 cm (width) × 8 cm (height). Each presentation mode included three stimuli conditions: *Same* (the target face and the test face were identical), *Composite* (the target and the test face had the same top half, but the bottom halves were different), and *Different* (the target face and test face were from two different persons) conditions.

##### 2.2.2.2 Procedures

**Figure 2:**
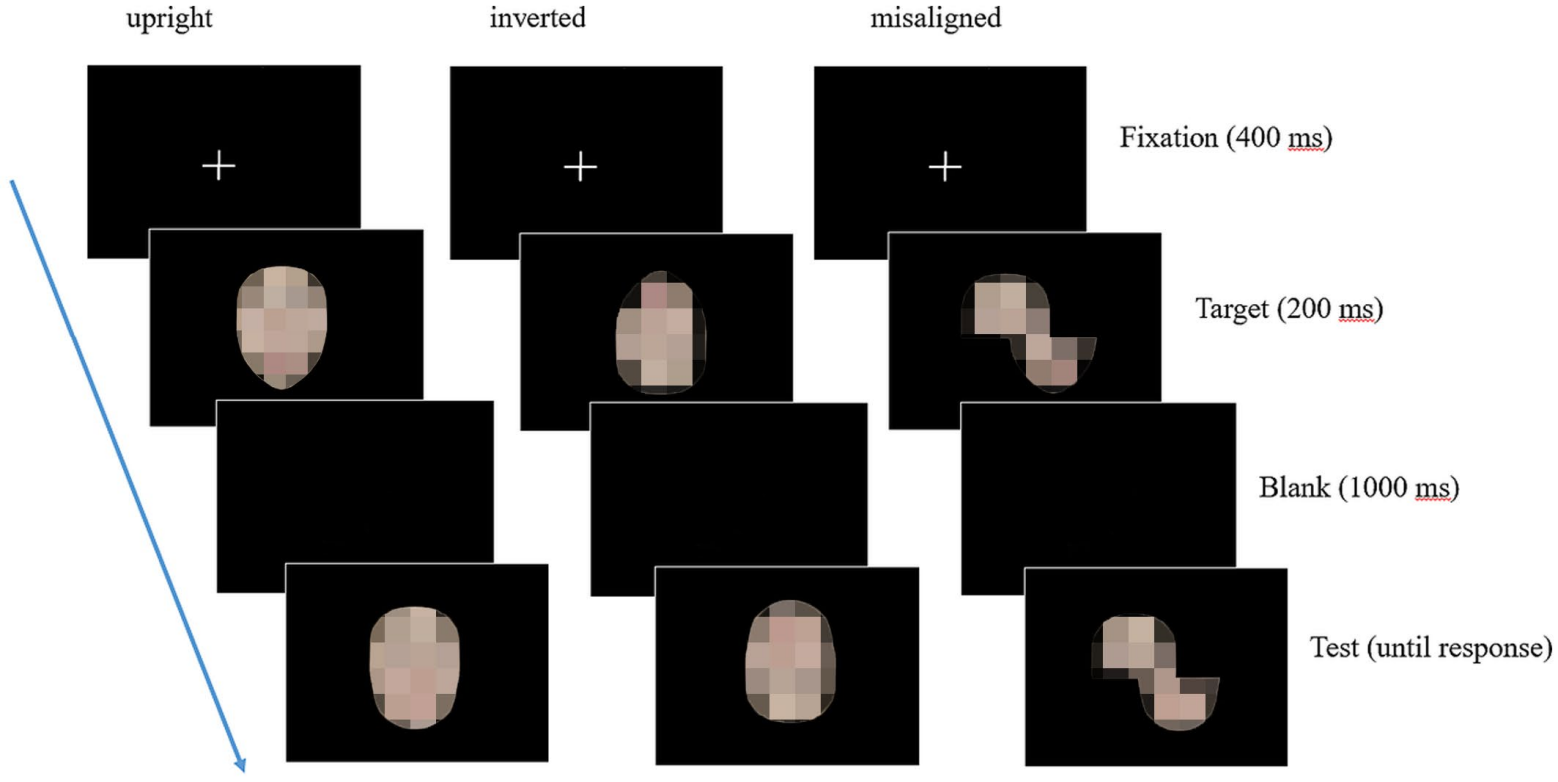
A sample trial of the composite face task for the three presentation modes. From left to right, the sample illustrates a “composite trial” condition (AB) for the aligned, the inverted, and the misaligned presentation modes. The identifiable images were blurred to protect the portrait copyright.

Figure 2 illustrates a sample trial of the composite face task for the three presentation modes. Each trial began with a fixation cross for 400 milliseconds (ms), followed by the presentation of the target face for 200 ms. After a 1,000 ms blank, the test face appeared on the screen and remained on until the participants made a response. The participants were instructed to judge whether the top half of the test face was the same as the top half of the target face by keypress responses. They were told to respond as quickly and accurately as possible. Each presentation mode was a separate block, presented in the fixed order of *aligned*, *inverted*, and *misaligned.* Each block included 48 trials (3 stimulus conditions × 8 sets of faces × 2 face genders) presented in randomized order (144 trials in total). The participants took a practice trial (using face stimuli not included in the formal experiment) at the beginning of each block to ensure they understood the task.

##### 2.2.2.3 Data preprocessing and statistical analyses

Each participant’s response accuracy and reaction time were recorded by E-Prime Professional 2.0. All statistical analyses were performed by SPSS 16.0 (SPSS, Inc., Chicago). Accuracy was indexed by the percentage of correct responses separately for the same, composite, and different trials at each presentation mode. Reaction Times (only for correct trials) was measured in the unit of millisecond (ms), separately for the same, composite, and different trials at each presentation mode. The outliers for Reaction Time measurements (i.e., ± 2.5 SD from the mean for each participant) were excluded from further analysis.

## 3 Results

### 3.1 The performance of the composite face task

The *accuracy* (ACC) and the *response time* (RT) were the two main dependent variables. To take both ACC and RT into account simultaneously to best capture the nature of task performances in both groups, we calculated the “*performance index*” in the form of ACC/RT^1^ (in seconds) of each stimuli condition in each presentation mode. This performance index will be used subsequently to analyze the magnitude of the composite face effects (CFE) (see Rossion, 2013, for a detailed review on p. 30, 31) and the face inversion effect (FIE). Table 2 illustrates the mean accuracies (ACC), the mean response times (RT), and the mean performance index (ACC/RT) for both groups of each stimuli condition at each presentation mode.

**Table 2:** The mean accuracy (ACC), mean response time (RT), and the mean performance index (ACC/RT) of the NT and ASD groups.

group	NT adults			ASD adults		
Presentation mode stimuli condition	Aligned	Inverted	Misaligned	Aligned	Inverted	Misaligned
ACC						
Same	0.80	0.79	0.84	0.78	0.79	0.82
	(0.04)	(0.03)	(0.04)	(0.03)	(0.03)	(0.03)
Composite	0.55	0.80	0.84	0.60	0.78	0.75
	(0.04)	(0.02)	(0.03)	(0.04)	(0.03)	(0.04)
Different	0.91	0.76	0.89	0.89	0.69	0.84
	(0.02)	(0.03)	(0.03)	(0.02)	(0.04)	(0.03)
RT(ms)						
Same	976	995	799	1,237	1,065	1,129
	(64)	(73)	(49)	(103)	(84)	(126)
Composite	1,210	949	874	1,434	1,096	1,105
	(95)	(64)	(64)	(133)	(72)	(124)
Different	927	1,062	918	1,108	1,183	1,147
	(50)	(63)	(62)	(70)	(97)	(104)
ACC/RT(s)						
Same	0.862	0.939	1.079	0.737	0.798	0.897
	(0.069)	(0.083)	(0.077)	(0.044)	(0.057)	(0.073)
Composite	0.611	0.961	1.103	0.526	0.819	0.838
	(0.074)	(0.090)	(0.080)	(0.045)	(0.063)	(0.079)
Different	1.006	0.859	1.076	0.813	0.749	0.912
	(0.060)	(0.061)	(0.056)	(0.046)	(0.072)	(0.068)

The numbers in the parentheses represent standard errors (SE) of the means.

A 3-way mixed ANOVA on the performance index ACC/RT was conducted with *Group* (NT, ASD) as the between-subject factor, *Presentation Mode* (aligned, inverted, misaligned) and *Stimuli Condition* (same, composite, different) as the within-subject factors. The *Group* main effect was significant, (*F*(1,46) = 2.905, *p* = 0.039, *η_p_^2^* = 0.089), the NT group performed better (*M* = 0.952, SE = 0.055) than the ASD group (*M* = 0.788, SE = 0.055), meaning that collapsed across conditions, the neurotypical adults tended to be more accurate and responded faster. The main effect of *Presentation Mode* was significant (*F*(2,92) = 26.307, *p* < 0.001, *η_p_^2^* = 0.364), the mean performances of *aligned*, *inverted*, and *misaligned* were 0.759 (SE = 0.036), 0.854 (SE = 0.044), 0.996 (SE = 0.048), respectively. With an adjusted error rate at *α* level = 0.05/3 = 0.016, the post-hoc Tukey HSD tests revealed that the mean performance index of the *misaligned* was significantly higher than that of the *aligned* (*p* < 0.001), but was not significantly different from that of the *inverted* (*p* = 0.027). The main effect of *Stimuli Condition* was also significant (*F*(2,92) = 7.927, *p* = 0.001, *η_p_^2^* = 0.147), the performance of *same*, *composite*, *different* were 0.885 (SE = 0.043), 0.810 (SE = 0.044), 0.914 (SE = 0.038) respectively. With an adjusted error rate at *α* level = 0.05/3 = 0.016, the mean performance index of the *different* was significantly higher than that of the *composite* (*p* = 0.005). Importantly, the Stimuli Condition* Presentation Mode interaction was significant (*F*(4,184) = 26.913, *p* < 0.001, *η_p_^2^* = 0.369); indicating that the difference in ACC/RT among the three stimuli conditions (the main effect of *Stimuli Condition*) varied across the three presentation modes. Likewise, the difference in ACC/RT among the three presentation modes (the main effect of *Stimuli Condition*) also varies across the three stimuli conditions.

**Figure 3:**
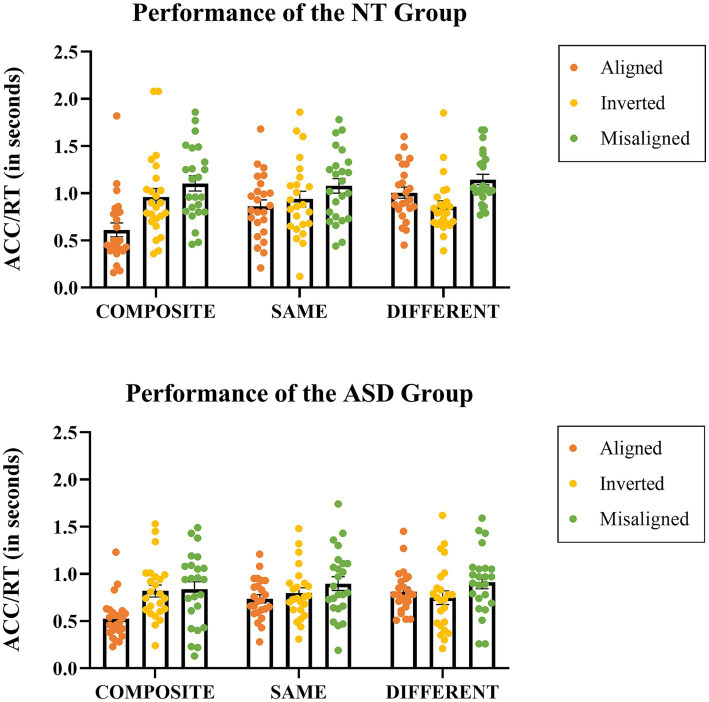
The performance indices in the form of Accuracy/Response time (in seconds) for each stimulus condition at each presentation mode in the NT group (top) and the ASD group (bottom). Each dot represents an individual data point, while the horizontal bars represent the group mean values.

We thus conducted further analyses comparing the three *Stimuli Conditions* (same, composite, different) on each *Presentation Mode* (aligned, inverted, misaligned) by three separate one-way ANOVAs. First, the one-way ANOVA on aligned mode with *Stimuli Condition* (same, composite, different) as the with-subject factor showed a significant main effect (*F*(2,46) = 50.508, *p* < 0.001), the mean scores of the same, composite, different conditions were 0.800 (SE = 0.041), 0.569 (SE = 0.043), and 0.910 (SE = 0.040), respectively. With an adjusted error rate at *α* level = 0.05/3 = 0.016, the mean score of the composite condition was significantly lower than that of the different condition (*p* < 0.001) and that of the same condition (*p* < 0.001). Second, the one-way ANOVA on inverted mode showed that the main effect of *Stimuli Condition* was not significant (*p* = 0.265), the mean scores of the same, composite, different conditions were 0.868 (SE = 0.051), 0.890 (SE = 0.055), and 0.804 (SE = 0.048), respectively. Lastly, the one-way ANOVA on misaligned mode showed that the main effect of *Stimuli Condition* was not significant either (*p* = 0.188), the mean scores of same, composite, and different were 0.988 (SE = 0.054), 0.970 (SE = 0.059), and 1.028 (SE = 0.047), respectively. In short, only the aligned (upright) mode exhibited a significant main effect of stimulus condition, while both the inverted and misaligned presentation modes did not (Figure 3).

### 3.2 Estimating the magnitude of holistic processing

To reveal whether the participants exhibited holistic face processing in the present tasks, we computed the differences in the performance index (ACC/RT) between the aligned and misaligned modes (i.e., the *composite face effect*), and the differences between the aligned and the inverted modes (i.e., the *face inversion effect*) for each participant.

#### 3.2.1 Misaligned minus aligned

We adopted Rossion (2013) formula to estimate the magnitude of the composite face effect as the consequences of spatial (mis)alignment based on the composite trials (i.e., identical top with a different bottom that is easily influenced by the different bottom-half to misjudge the top-face as different) as follows:


$$\begin{array}{l} \mathrm{Misaligned}\;\mathrm{minus}\;\mathrm{Aligned}\\ =\mathrm{performance index of the}\;\mathrm{misaligned}\;\mathrm{condition}\\ -\mathrm{performance index of the}\;\mathrm{aligned}\;\mathrm{condition} \end{array}$$

**Figure 4:**
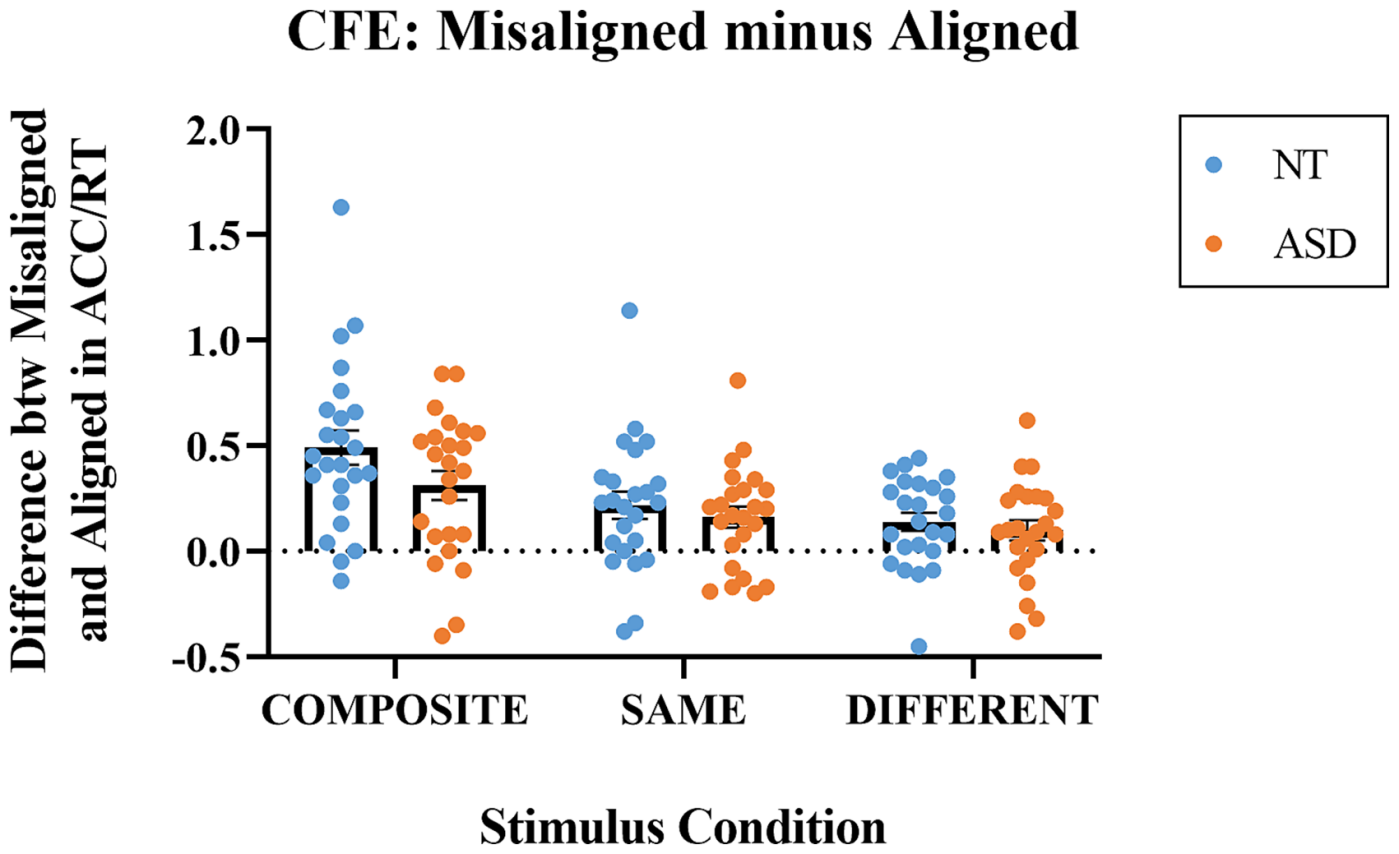
The indices of Misaligned minus Aligned (differences in ACC/RT) for the composite, same, and different stimulus conditions. Blue dots represent the individual data for the NT group; orange dots represent the individual data for the ASD group. The horizontal bars represent the group mean values.

Figure 4 illustrates the differences in the performance index (ACC/RT) between the aligned and misaligned modes at the three stimulus conditions for both groups. The most interesting condition type is the composite trials, and a value significantly greater than zero indicates the presence of the *composite face effect* (i.e., evidence for holistic processing). For the composite condition, the mean indices were 0.490 (SE = 0.081) for the NT group and 0.312 (SE = 0.069) for the ASD group. Both indices were significantly higher than zero (NT: *t*(23) = 6.036, *p* < 0.001; ASD: *t*(23) = 4.496, *p* < 0.001). For the same condition, the mean indices were 0.217 (SE = 0.064) for the NT group and 0.161 (SE = 0.050) for the ASD group. Both indices were significantly higher than zero (NT: *t*(23) = 3.384, *p* = 0.003; ASD: *t*(23) = 3.226, *p* = 0.004). For the different condition, the mean indices were 0.139 (SE = 0.043) for the NT group and 0.098 (SE = 0.048) for the ASD group. Both indices were also significantly greater than zero (NT: *t*(23) = 3.207, *p* = 0.004; ASD: *t*(23) =2.084, *p* = 0.048).

We conducted a 2-way mixed ANOVA on the differences in ACC/RT (misaligned –aligned) with *Group* as the between-subject factor and *Stimulus Condition* (same, composite, different) as the within-subject factor. The *Group* main effect was not significant (*p* = 0.212) the mean of NT group (*M* = 0.261, *SE* = 0.037) was higher but not significantly different from that of ASD group (*M* = 0.183, *SE* = 0.033). The main effect of *Stimulus Condition* was significant (*F*(1,46) = 36.872, *p* < 0.001, *η_p_^2^* = 0.445); the means of the composite trials, same trials, and the different trials were 0.401 (*SE* = 0.053), 0.189 (*SE* = 0.041), and 0.119 (SE = 0.032), respectively. With an adjusted error rate at *α* level = 0.05/3 = 0.016, the CFE index for the composite trials was significantly higher than those for the same trials (*p* < 0.001) and the different trials (*p* < 0.001); the CFE index for the same trials was slightly higher than that for the different trials (*p* = 0.042). The Group * Composite Type interaction effect was not significant (*p* = 0.145). To sum up, both the NT and ASD groups exhibited greater-than-zero *composite face effects* that their performance enhanced in the misaligned presentation mode and the magnitude was qualitatively similar in both groups.

#### 3.2.2 Inverted minus aligned

Likewise, we adopted a conceptually similar formula to estimate the magnitude of the face inversion effect to see whether inversion effectively disrupted the holistic processing as follows. We also expected that the inversion would exert the opposite influence for the composite trials and the different trials.


$$\begin{array}{c} \mathrm{Inverted}\;\mathrm{minus}\;\mathrm{Aligned}=\mathrm{performance index of the}\;\mathrm{inverted}\;\mathrm{condition}\\ -\mathrm{performance index of the}\;\mathrm{aligned}\;\mathrm{condition} \end{array}$$

**Figure 5:**
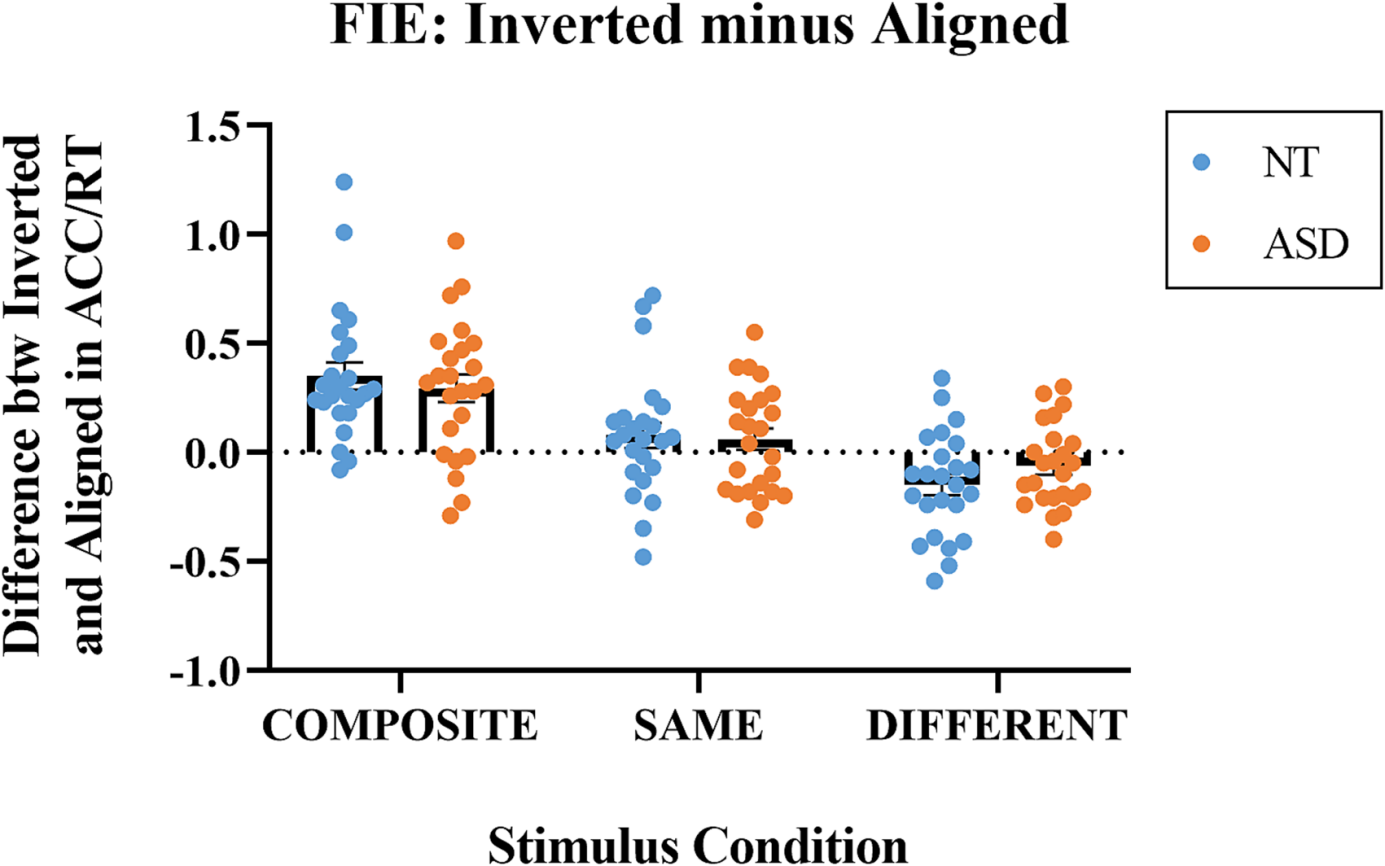
The indices of Inverted minus Aligned (differences in ACC/RT) for the composite, same, and different stimulus conditions. Blue dots represent the individual data for the NT group; orange dots represent the individual data for the ASD group. The horizontal bars represent the group mean values.

Figure 5 illustrates the differences in the performance index (ACC/RT) between the inverted and aligned modes at the three stimulus conditions for both groups. For the composite condition, the mean indices were 0.351 (SE = 0.062) for the NT group and 0.293 (SE = 0.063) for the ASD group. Both indices were significantly higher than zero (NT: *t*(23) = 5.676, *p* < 0.001; ASD: *t*(23) = 4.617, *p* < 0.001). A value significantly greater than zero indicates that the inversion effectively interrupted holistic processing; hence, the illusion created by the different bottom halves was eliminated when the faces were presented upside down (i.e., better performances in the inverted condition). For the same condition, the mean indices were 0.077 (SE = 0.058) for the NT group and 0.059 (SE = 0.049) for the ASD group. Both indices were not significantly from zero (NT: *t*(23) = 1.324, *p* = 0.199; ASD: *t*(23) = 1.231, *p* < 0.231). For the different condition, the mean indices were − 0.1483 (SE = 0.049) for the NT group and − 0.064 (SE = 0.038) for the ASD group. The NT group, but not the ASD group, was significantly deviated from zero (NT: *t*(23) = −3.033, *p* = 0.006; ASD: *t*(23) = −1.661, *p* = 0.105). The ASD group was marginally lower than zero If we adopted a one-tailed *t*-test (*p* = 0.052).

We conducted a 2-way mixed ANOVA on the differences in ACC/RT (inverted –aligned) with *Group* as the between-subject factor and *Stimulus Condition* (same, composite, different) as the within-subject factor. The *Group* main effect was not significant (*p* = 0.959); the mean of NT group (*M* = 0.086, SE = 0.040) was not significantly different from that of ASD group (*M* = 0.092, SE = 0.033). The main effect of *Stimulus Condition* was significant (*F*(1,46) = 64.645, *p* < 0.001, *η_p_^2^* = 0.584); the means of the composite trials, same trials, and the different trials were 0.322 (SE = 0.044), 0.068 (SE = 0.038), and − 0.106 (SE = 0.031), respectively. With an adjusted error rate at *α* level = 0.05/3 = 0.016, the FIE index for the composite trials was significantly higher than those for the same trials (*p* < 0.001) and the different trials (*p* < 0.001); the FIE index for the same trials was significantly higher than that of the index for the different trials (*p* < 0.001). The Group * Composite Type interaction effect was not significant. To sum up, both the NT and ASD groups exhibited significant face inversion effects based on the composite trials. Notably, face inversion indeed exerted the opposite influence for the composite trials (i.e., better performances when inverted) and the different trials (i.e., worse performances when inverted) for both groups and with similar magnitudes.

### 3.3 Correlations between the two indices and the AQ score

**Figure 6:**
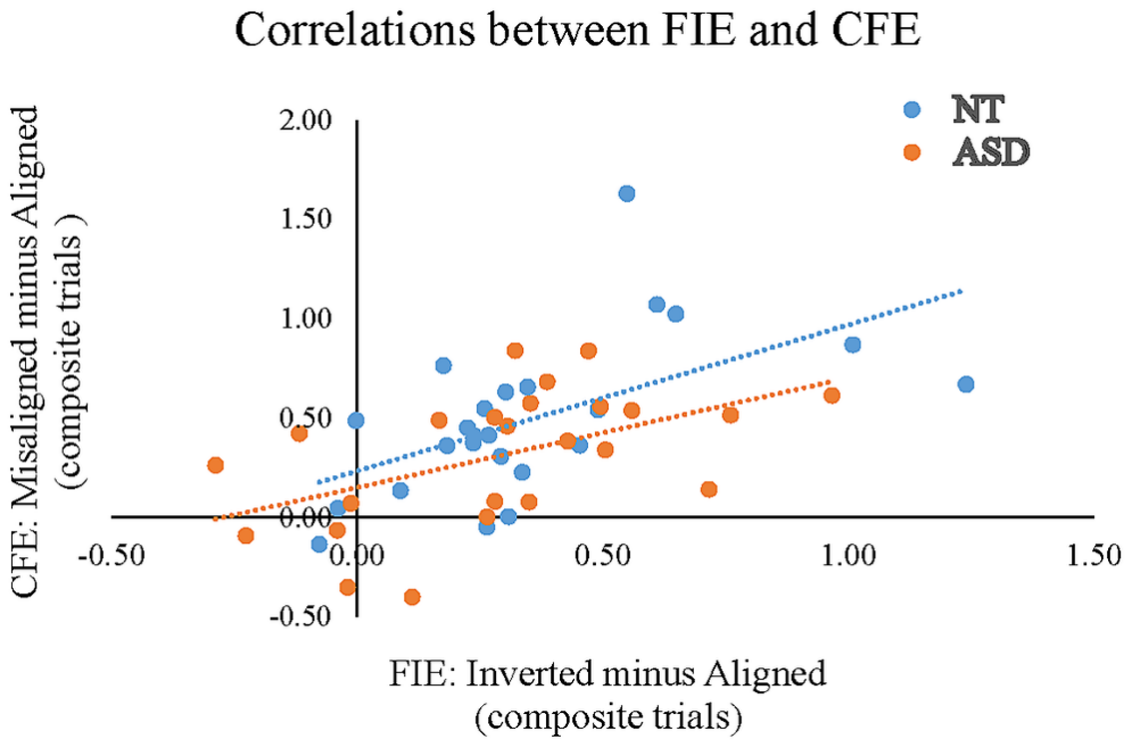
Pearson’s correlations between the FIE based on the composite trials of the “*Inverted minus Aligned*” (the X-axis) and the CFE based on the composite trials of the “*Misaligned minus Aligned*” in ACC/RT (the Y-axis) for the NT and ASD groups. The blue dots (and line) represent the neurotypical adults; orange dots (and line) represent the individuals with ASD.

To reveal whether the magnitude of the composite face effect (CFE) correlated with the magnitude of the face inversion effect (FIE) in neurotypical adults and adults with ASD, we conducted two separate Pearson’s bivariate correlation analyses between AQ scores and the index of “*Misaligned minus Aligned*” and the index of “*Inverted minus Aligned*” in ACC/RT for the NT and the ASD groups. Figure 6 illustrates the correlations between the CFE and the FIE. For the NT group, the individuals’ magnitude of FIE positively correlated with the CFE (*r* = 0.563, *p* = 0.004), indicating that individuals with a greater FIE tended to show a larger composite face effect. For the ASD group, the individuals’ magnitude of FIE also positively correlated with the CFE (*r* = 0.503, *p* = 0.012).

**Figure 7:**
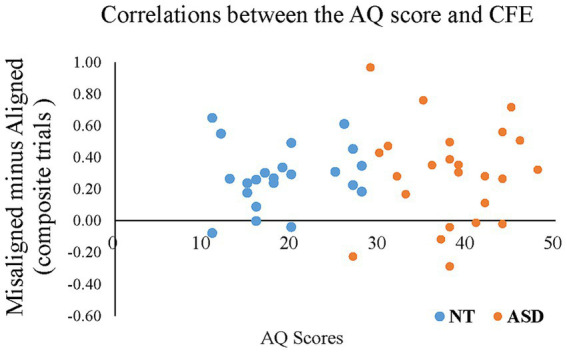
The Pearson’s correlations between individual AQ scores (the X-axis) and the CFE based on the composite trials of “*Misaligned minus Aligned*” in ACC/RT (the Y-axis) for all participants. The blue dots represent the neurotypical adults; orange dots represent the individuals with ASD.

To reveal whether the magnitude of the composite face effect correlated with an individual’s AQ score, we conducted Pearson’s correlation between AQ scores and the CFE index of “*Misaligned minus Aligned*” in ACC/RT for all participants. Figure 7 illustrates the correlation between the AQ score and the magnitude of CFE. As expected, the individuals’ AQ scores negatively correlated with the CFE (*r* = −0.285, *p* = 0.049), indicating that individuals with higher autistic traits tended to show less holistic processing (i.e., a smaller composite face effect). The correlation between the AQ score and the magnitude of FIE did not reach statistical significance.

## 4 Discussion

Using a custom-designed composite face task, the present study investigated holistic face processing in neurotypical adults and adults with ASD by assessing the magnitude of face inversion effect (FIE) and composite face effect (CFE) simultaneously and independently. Several findings are noteworthy: First, there is a significant group difference in the overall performance (in ACC/RT); the NT group tended to be more accurate in less time as compared to the ASD group. Second, both the NT adults and adults with ASD exhibited a greater–than–zero composite face effect (CFE), suggesting that both groups exhibited holistic processing manifested by the effect of spatial (mis)alignment when viewing chimeric faces. Third, both the NT and ASD groups showed a significant face inversion effect (FIE) with similar magnitude, indicating that both groups used holistic processing when viewing upright faces. Fourth, the correlations further revealed that, for all participants, the individuals’ magnitude of FIE positively correlated with the magnitude of CFE, indicating that those who exhibited a gre ater face inversion effect also tended to show a larger composite face effect. Last but not least, a mild negative correlation between the AQ scores and the magnitude of CFE was observed, meaning that individuals with lower AQ scores tended to exhibit a slightly larger illusion induced by the composite faces. In summary, our findings suggest that adults with ASD exhibit holistic processing in a qualitatively similar way as neurotypical adults when viewing faces.

### 4.1 The ASD group exhibited a significant face inversion effect similar to the NT group

Our study demonstrated that the ASD group exhibited a significant face inversion effect just like the NT group, manifested by the greater-than-zero index of *Inverted minus Aligned*, and that face inversion exerted opposite effects for the composite and the different trials. In the case of the composite trials, both groups performed better in the inverted condition (i.e., positive values), meaning that the inversion effectively interrupted holistic processing; hence, the composite illusion created by the different bottom halves was eliminated when the faces were presented upside down (i.e., better performances in the inverted condition). This particular finding echoes the early study of Hole (1994) and Hole et al. (1999), who also used an inverted-aligned presentation mode as a control condition and reported that inversion could improve the performance of the composite trials (also see Rossion, 2013). Even more interesting is the case of different trials—the top and bottom halves of the target face and the test faces are from different identities. The face inversion effect under such stimulus condition is very similar to the typical inversion effect in which the performance was better when the faces were upright and declined when presented upside-down (i.e., negative values). As revealed in Figure 5, although only the value of NT group was significantly less than zero, the value of ASD group was also negative (i.e., *p* = 0.052, marginally different from zero for a one-tail *t*-test), showing a similar pattern of responses as the NT group.

How can we reconcile the present results with recent findings showing a reduced FIE in adults with ASD (Hadad et al., 2019; Hartston et al., 2023)? Using a morphing paradigm, Hadad et al. (2019) suggest that adults with ASD do not attain enough face processing expertise to process frequent-race faces (i.e., own-race face) in a different manner than less-frequent-race faces (i.e., other-race face). While TD individuals exhibited significantly greater inversion effects (with a robust, specific face marker typically seen for own-race faces), adults with ASD showed overall lower and comparable inversion effects for own- and other-race faces. One explanation could be that the present study adopted the performance index (i.e., ACC/RT) in which the tradeoff between accuracy and RT was taken into account. If we had computed the differences in accuracy only, we might have observed a reduced magnitude of FIE, as suggested by Hadad et al. (2019).

Nevertheless, our findings are consistent with studies indicating that autistic adults showed a reliable face inversion effect similar to NT adults. For instance, Suzanne Scherf et al. (2008) found that both the typically developing children and adults and the ASD group exhibited reliable face inversion effects, and the three groups did not differ much in the size of the inversion effect. Lastly, Teunisse and de Gelder (2003) showed that their results on the inversion task suggested that most autistic adolescents can form a normal configuration-based face representation. However, the absence of the composite effect indicates that autisitc adolescents are less prone to use the contextual information of the face in a visual-search task. In short, the presence of significant FIE in both groups as well as the absence of group differences challenges the view that facial processing is atypical in ASD, suggesting that autistic individuals also engage in configural processing to perceive whole faces.

### 4.2 Both the TD and the ASD groups showed significant composite face effects

Notably, both the TD and the ASD groups exhibited significant composite face effects (CFE), manifested by the greater-than-zero indices of *Misaligned minus Aligned* for all three stimulus conditions, as shown in Figure 4. We expected that the composite trials--the most interesting condition type that shall benefit the most from the spatial misalignment to break up the influence of holistic processing—to be significantly greater than zero. Indeed, both groups exhibited positive values; the magnitude of the ASD group was slightly smaller, but it was not statistically different from that of the NT group. Interestingly, indices of *Misaligned minus Aligned* for the same and the different trials were also significantly greater than zero, and the magnitude was much smaller than that of the composite trials. We did not expect to see a composite face effect for the same trials (i.e., the two identical top halves are aligned or misaligned with identical bottom halves) because the performance for trials in which the bottom half does not change shall not be much influenced by the spatial alignment of the two halves. Likewise, we did not expect to see a composite face effect for the different trials either (i.e., the two different top halves are aligned or misaligned with different bottom halves). However, the small but significantly greater than zero effect of these two conditions may reflect a generally detrimental influence due to the spatial alignment. Nevertheless, it is important to highlight that ASD and NT groups exhibited a very similar pattern of results for all three types of trials, indicating intact holistic processing in adults with ASD.

Our findings deviated from the results reported by Teunisse and de Gelder (2003), they noted that the ASD group did not show the composite face effect as the TD group did. The ASD group recognized the aligned composite faces and the non-aligned composite faces equally well. The discrepancy between our findings and theirs could be due to the drastic differences in the task manipulations. In Teunisse and de Gelder (2003), the target was in front view, but the test was in 3/4 view, and both the same and different trials (Exp1) and the same and composite trials (Exp2) were presented simultaneously in the test display. In their Exp 2, although they had a complete composite task (change both top halves and bottom halves), they did not include the different trials (change to another person) as a control baseline condition. Another possible confounding factor is age; Teunisse and de Gelder (2003) and Gauthier et al. (2009) tested adolescents with ASD and found a reduced or atypical composite face effect; however, our study tested adults with ASD and found a clear presence of the composite face effect. It could be that holistic processing was deviant by adolescence but becomes more typical (i.e., quantitatively different) by adulthood. In support of this ‘developmental delay hypothesis,” several previous studies focusing on adults with and without ASD (Nishimura et al., 2008; Weigelt et al., 2012; Ventura et al., 2017; but see Tang et al., 2015) also reported the presence of CFE. For example, Nishimura et al. (2008) found that the ASD group demonstrated normal holistic processing (i.e., showing composite face effect), normal sensitivity to second-order relations in upright faces, and the expected disruption of sensitivity to second-order relations in inverted faces. Likewise, using two versions of the composite face task (i.e., the complete composite task design and the VHFPT2.0 paradigm), Ventura et al. (2017) demonstrated that autistic adults process face holistically with the same efficiency as typical adults.

### 4.3 The presence of positive correlations between the CFE and FIE in all participants

Three measures are commonly used to quantify the face-specific “holistic processing”: the face inversion effect (FIE), the part-whole effect, and the composite face effects (CFE). The face inversion effect takes the difference in performance between upright and inverted faces. The composite face effect takes the difference in performance between the aligned and the misaligned faces. The present study examined a central question of whether both measures of holistic processing, the FIE and the CFE, tap into the same perceptual mechanisms. The widespread assumption in the face perception literature is that they do. Although Rezlescu et al. (2017) found that the inversion and part-whole effects were only modestly correlated and that the composite effect did not correlate with either, other studies reported moderate correlations and considered that the three effects (the FIE, the CFE, and the part-whole effect) are different ways to measure the same phenomenon (Behrmann et al., 2015; Duchaine and Yovel, 2008; McKone et al., 2007; Piepers and Robbins, 2012; Rhodes, 2013; Tanaka and Gordon, 2011). The present findings are consistent with this assumption, as we observed that the individuals in either the NT or the ASD group exhibited a significant positive correlation between the composite face effect (CFE) and face inversion effect (FIE), as elucidated in Figure 6. In other words, the correlational analyses provided evidence that both measures seem to tap into the same mechanism of holistic processing within an individual.

## 5 Conclusion, limitation, and future work

In summary, the present study delved into an important question about whether individuals with ASD use holistic face processing in a qualitatively similar way as typical adults. By assessing the magnitude of the face inversion effect (FIE) and composite face effect (CFE) simultaneously and the strength of correlations between the two effects, we demonstrated that autistic adults exhibited holistic processing when viewing faces. These findings are consistent with Nishimura et al. (2008), Weigelt et al. (2012) and Ventura et al. (2017), that autistic individuals exhibited normal holistic processing, and their facial identity processing abilities were qualitatively similar to typical adults. Some limitations of the present study include that our task only focused on the top-half face; we would like to include tasks that also involve judging the same/differences of the bottom half for future work. Secondly, although we matched both groups based on their years of education, we could not assess participants’ IQ levels in the current study. However, we are in a good position to say that the current results are unlikely due to IQ differences because neither CFE nor FIE exhibited a significant group difference. Last but not least, although we presented evidence of holistic processing in autistic adults, this does not imply that they have exactly the same face-processing capacities as neurotypical adults. Adopting multiple tasks and eye-tracking devices to further explore the pattern of eye fixation and pupillometry (Falck-Ytter, 2008) can be an important next step to reveal the processing characteristics in autistic individuals.

## Data Availability

The raw data supporting the conclusions of this article will be made available by the authors, without undue reservation.

## Footnotes

^1^In our preliminary analyses, which looked at only accuracy or RT, we did find that the group differences in accuracy and response time were in the same direction (the autistic group tended to make more errors and respond more slowly). However, the effect from each separate measurement was small. We found that using the ACC/RT performance index can augment the effect and better reveal the difference.
